# The effect of transcranial Direct Current Stimulation on the Iowa Gambling Task: a scoping review

**DOI:** 10.3389/fpsyg.2024.1454796

**Published:** 2024-12-18

**Authors:** Silvia Salice, Alessandro Antonietti, Laura Colautti

**Affiliations:** Department of Psychology, Università Cattolica del Sacro Cuore, Milan, Italy

**Keywords:** decision making, Iowa Gambling Task, transcranial direct current stimulation, dorsolateral prefrontal cortex, orbitofrontal cortex

## Abstract

**Introduction:**

Among the tasks employed to investigate decisional processes, the Iowa Gambling Task (IGT) appears to be the most effective since it allows for deepening the progressive learning process based on feedback on previous choices. Recently, the study of decision making through the IGT has been combined with the application of transcranial direct current stimulation (tDCS) to understand the cognitive mechanisms and the neural structures involved. However, to date no review regarding the effects of tDCS on decisional processes assessed through the IGT is available. This scoping review aims to provide a comprehensive exploration of the potential effects of tDCS in enhancing decisional processes, assessed with the IGT, through the evaluation of the complete range of target cases.

**Methods:**

The existing literature was analyzed through the PRISMA approach.

**Results:**

Results reported that tDCS can enhance performance in the IGT and highlighted a pivotal role of the dorsolateral prefrontal cortex and the orbitofrontal cortex in risky and ambiguous decisions.

**Discussion:**

Thus, tDCS over the brain regions identified improves the decisional processes in healthy subjects and patients, confirming its potential to enhance decision making in everyday contexts and deepen the neural correlates. Suggestions for further studies are provided to delve into decisional mechanisms and how to better support them.

## 1 Introduction

Decision making (DM) is a complex cognitive process that plays a crucial role in everyday contexts, covering various areas in people’s lives (Lannello et al., 2017; Colautti et al., 2022). Generally, making a decision involves reasoning under conditions of uncertainty since it is not possible to predict the outcome or the consequences of a choice. In this way, DM situations can be categorized based on the probability of outcomes related to the available alternatives in two main conditions: DM under ambiguity and DM under risk. Ambiguity involves situations where the probability of positive or negative outcomes associated with at least one option is unknown, while risk involves situations where the probabilities for each possible consequence are known, presenting a higher number of data to be considered throughout the decisional process (Bechara and Martin, 2004; Brand et al., 2007; Lauriola et al., 2007; Colautti et al., 2022).

From a cognitive perspective, DM requires several steps, which can be influenced by emotions triggered by the situation. These steps include recognizing the current situation and identifying crucial information, continuously updating available information, evaluating possible outcomes according to personal goals and motivation, inhibiting impulsive but not appropriate responses, anticipating the possible consequences of the alternatives based on available data and previous feedback, making a decision in line with personal goals, and re-evaluating the decision based on the outcome (e.g., Rangel et al., 2008). Thus, to delve into decisional processes, it is crucial considering that both the cognitive and the affective components have a role. In this way, the Iowa Gambling Task (IGT; Bechara et al., 1994) can be particularly useful and is the most frequently used task to investigate DM involving ambiguity and risk, as it allows to assess the ability to identify risk–benefit relationships and to sacrifice short-term gains over long-term benefits (Buelow, 2015).

In this task, the player must select a card from four decks (A, B, C, and D) for 100 times (or trials). Each choice produces a monetary gain or loss, according to the features of the selected deck. Two decks (usually A and B) are designed to be “disadvantageous” as they are associated to high wins but also inflict higher losses (resulting in a long-term negative result), whilst the other two decks (C and D) are “advantageous” as they provide lower wins than the prior ones, but also lower losses (guaranteeing a successful long-term result). At the beginning of the task, players do not have any information regarding the features of the decks, but they are only told that their goal is to maximize benefits and minimize losses. In each trial, a win or loss feedback is provided to the player immediately after making every choice and so, as the task progresses, the participant will be able to consider more data in order to make his/her choices. To measure the decisional performance through the IGT, the total net score is usually considered. It is obtained by making the difference between the number of choices of advantageous decks minus the number of choices of disadvantageous decks [(C + D)–(A + B)] (Danner et al., 2012): Higher net scores indicate a higher number of advantageous choices and so a more functional task performance. Other parameters used in some studies to better investigate the performance are the blocks’ net score, which is the net score computed over blocks of trials [usually 5 of 20 trials or 2 of 50 trials (e.g., Colautti et al., 2021)] and is useful to investigate the presence of a learning process, based on feedbacks during the task, if in the last blocks the net scores are higher than in the first ones. As well, the amount of money earned/lost at the end of the game has been considered by some authors, where a total negative income is assumed to be indicative of a risky attitude.

The IGT involves two kinds of cognitive operations: learning about the task structure from the cards’ feedback and using information gained from previous feedbacks to determine which deck to select from (Bowman and Turnbull, 2004; Stocco et al., 2009). This involves the interplay between learning outcomes and applying the acquired knowledge to the decisional process (Stocco et al., 2009). Regarding the learning process, the task can be useful to delve into the mechanisms underlying the transition from DM under ambiguous conditions to DM under risk, a shift which usually occurs during a standard 100-choice interaction along the IGT in case of unimpaired cognitive and decisional abilities (Brevers et al., 2013; Stocco et al., 2009). In fact, to make gainful choices along the task, the players need to select the two advantageous decks out of the four presented, structuring a correct mental representation of the decks based on the feedback received for each trial. Although during the first trials the player is usually not able to identify which are the advantageous decks (and therefore, choices occur in a situation of predominant ambiguity), after a certain number of feedbacks the player should progressively learn to discern more precisely which decks are advantageous or not (framing a condition of choice under risk since the outcomes’ probability and entity for each decision are more outlined; e.g., Brand et al., 2007; Buelow et al., 2014; Ko et al., 2010). This peculiarity allows researchers to investigate whether the players are able to create correct representations—stable over time regardless of the single immediate result—of the options based on feedback (Brand et al., 2007; Colautti et al., 2022; Simonovic et al., 2018). In this way, a higher rate of risky decisions is assumed to mainly underlie failures (i) in correctly representing advantageous and disadvantageous decks along the task and/or (ii) in planning advantageous strategies for long-term benefits, shifting from immediate rewards to delayed cumulative gains (Antonietti et al., 2023; Colautti et al., 2021). Impairments in the progressive awareness of the features of the decks and in shifting from choosing the disadvantageous ones to selecting the advantageous one are frequently associated with behavioural disorders, such as pathological gambling (Soyata et al., 2019) and substance-abuse (Li et al., 2010), or neurological conditions, such as Parkinson’s disease (PD), that involve neural structures pivotal for the decisional process (Brand et al., 2004; Colautti et al., 2023).

Making an advantageous choice requires a synergic interaction between “cold” aspects of cognition, such as the regulation and optimization of goal-directed behaviours while countering automaticity (through inhibition of responses no longer appropriate, planning and making previsions, updating, set-shifting, and monitoring: Costafreda et al., 2006; Hampshire et al., 2010; Aron et al., 2014; Friedman and Robbins, 2022), and “hot” aspects, involving affective and motivational components, incentive-values, and/or avoidance-tendencies (Salehinejad et al., 2021). Accordingly, during the IGT, neuroimaging studies highlighted the activation of crucial neural structures such as:

- the dorsolateral prefrontal cortex (DLPFC), that underpins operations requiring high-order cognitive abilities encompassing cold executive functions (EFs) such as updating processes, cognitive flexibility, and strategic planning;
- the orbitofrontal cortex (OFC), crucial in processing the value of a stimulus based on feedback, elaborating emotionally charged events (as it can be a monetary gain or loss), and updating stimulus–reward associations;
- the anterior cingulate cortex (ACC), associated with motivation behaviours, encoding choice value, conflict detection, and error monitoring, in addition to subcortical structures such as the ventral striatum and the insula, associated with motivation, emotions, and prediction of rewards (Chau et al., 2018; Foerde and Shohamy, 2011; Li et al., 2010).

To delve into the mechanisms and to understand the role of neural bases involved in the decisional processes, researchers applied different methodologies, including the transcranial direct current stimulation (tDCS), which produces changes in spontaneous neuronal excitability in the cerebral cortex through the application of weak electrical currents to the scalp. This generates a modulation of the excitability of the neurons with two different modalities. The anodal stimulation usually creates an excitatory effect through the depolarization of the stimulated brain area, whereas the cathodal stimulation leads to an inhibitory result through the hyperpolarization of the neurons (Strobach and Antonenko, 2017). The adoption of IGT to investigate the stages and DM processes is a safe procedure—as it does not typically cause serious adverse effects—and can be extended to various population targets, encompassing both healthy subjects and patients with neurological or behavioural pathologies (Prehn and Flöel, 2015; Stagg and Nitsche, 2011).

In this way, tDCS seems useful for (i) investigating the neurophysiological bases of behavioral and psychological processes, providing insights into the crucial mechanisms underlying DM and (ii) better understanding how to modulate and enhance decisional performance. Even if such a piece of knowledge can be important to design effective therapeutic interventions, to date there is a lack of studies that analyze and summarize evidence in literature about the effects of tDCS on the IGT considering different target populations (e.g., Shin et al., 2015).

Although tDCS provides several advantages to the analysis of DM, it is important to discuss some technical limitations. A first issue regards the localization of currents’ action in the brain and the prediction of its impact on cognitive abilities (Buelow, 2015). More precisely, this point concerns tDCS’ limited spatial accuracy: As the current passes through the brain from anode to cathode and modulates neural activity, it can be difficult to relate the effects of tDCS to a specific brain region (Prehn and Flöel, 2015). Moreover, it can be challenging to determine if the tDCS has been applied with sufficient precision to the targeted cortical regions (Westwood et al., 2017). Other issues concern the impossibility to confirm if equal or similar amounts of current reach all brain areas of each participant in terms of individual variation, or the actual directionality of tDCS effects within the brain (van’t Wout and Silverman, 2017). While reversing polarity often leads to opposite effects on cognitive processes, this is not always consistent (Das et al., 2016). Furthermore, there may be differences in modulating cognition through tDCS in brains affected by pathologies that display a reduced or impaired neuronal excitability or processing capacity and in healthy brains with optimal functioning levels (Westwood et al., 2017). So far, the underlying neural mechanisms of tDCS still need to be fully uncovered (Pacheco-Barrios et al., 2020). Finally, some ethical questions have been raised, including under what circumstances the use of tDCS is justifiable and appropriate in the contexts of both therapy and cognitive enhancement (Cancer et al., 2021; Day et al., 2022).

Nevertheless, considering the potential of this technique, the present review aims at identifying findings concerning the effects of tDCS on the decisional performance involving ambiguous and risky conditions assessed through the IGT. The scoping review modality was adopted to provide a comprehensive analysis of the available findings, to identify possible gaps in the existing literature, and to provide possible useful suggestions for further studies (Arksey and O’Malley, 2005).

## 2 Materials and methods

**Figure 1:**
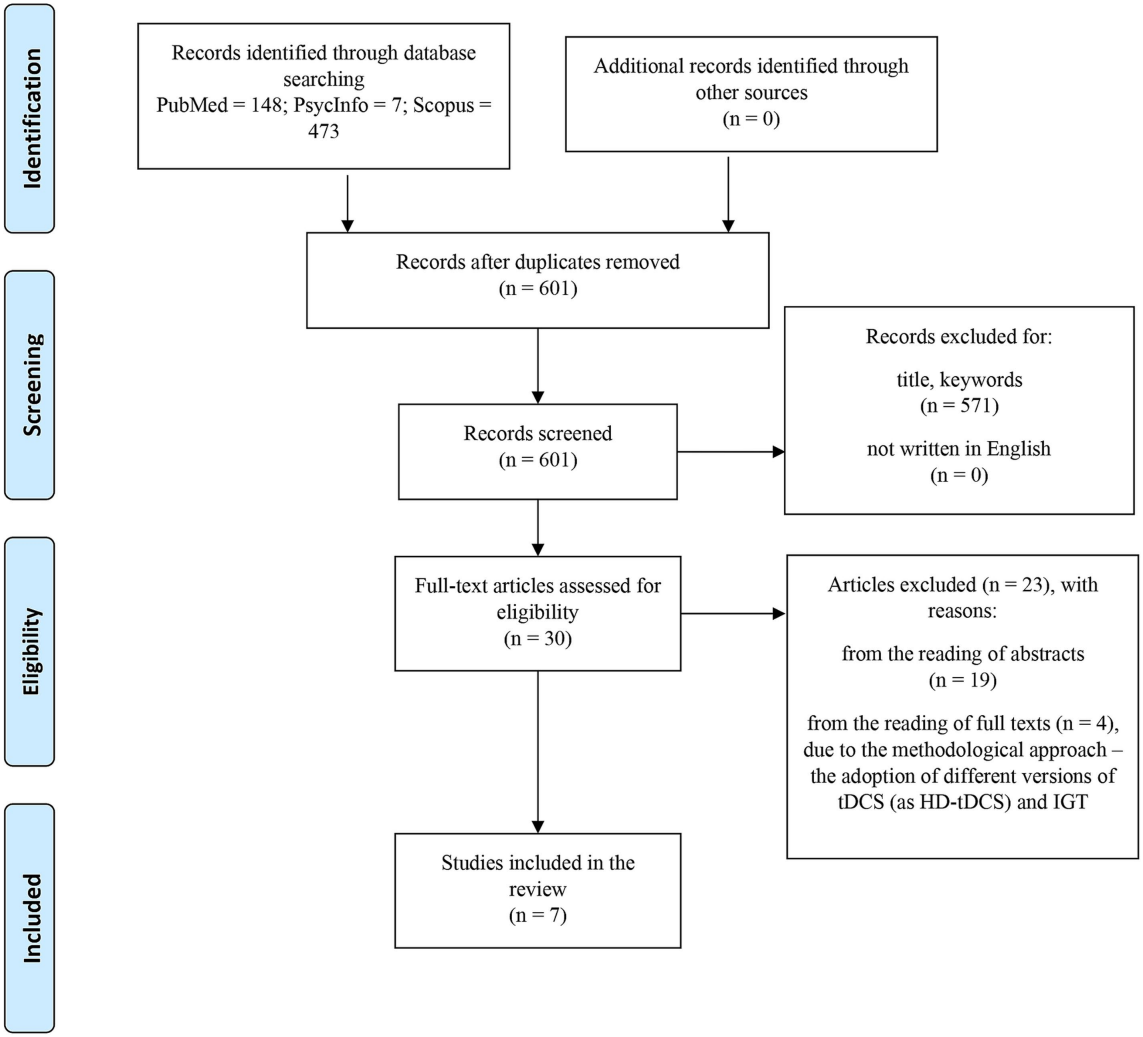
Process of identification and screening for included articles.

The current scoping review was drafted according to the Preferred Reporting Items for Systematic Reviews and Meta-Analysis Extension for Scoping Reviews (PRISMA-ScR; Tricco et al., 2018; see Figure 1). The five-stage framework (Arksey and O’Malley, 2005) was adopted to design the present review, in order to (i) increase the reliability and the replicability of the findings, (ii) eliminate any gaps in methodological rigor, and (iii) ensure in-depth and broad results.

Specifically, the five stages include:

1. identifying the research question;
2. identifying relevant studies;
3. study selection;
4. charting the data;
5. collating, summarizing, and reporting the results.

### 2.1 Identifying the research question

The present scoping review focuses on two main questions: “Does tDCS lead to an improvement in risky and ambiguous DM assessed through the IGT in both healthy population and clinical conditions?” and “Does anodic tDCS has a higher influence on the DM process than cathodal tDCS or is preferable to design a coupled anodal and cathodal tDCS?”

### 2.2 Identifying relevant studies

The search began in December 2023. An examination of the following bibliographic databases was conducted in order to identify relevant peer-reviewed articles: Scopus, PsycINFO, and PubMed. Based on the aims of the search, the keywords entered in the search engine for each database previously specified were as follows: “Iowa Gambling Task AND (tDCS),” “Iowa Gambling Task” AND (tES),” “Iowa Gambling Task” AND (neurostimulation),” “Iowa Gambling Task” AND (neuromodulation),” “IGT AND (tDCS),” “IGT AND (tES),” “IGT AND (neurostimulation),” “IGT AND (neuromodulation),” “Transcranial Direct Current Stimulation” AND (Iowa Gambling Task), “Transcranial Direct Current Stimulation” AND (IGT), “Transcranial Direct Current Stimulation” AND (decision making), “tDCS” AND (decision making). After the study selection (Stage 3, see below), bibliographies belonging to the selected articles were checked to verify other potential eligible studies.

Specific inclusion criteria were applied, following the PICO approach (da Santos et al., 2007): (1) studies involving adult participants (≥ 18 years old); (2) studies applying tDCS and adopting the IGT as the primary task to investigate DM; (3) the standard version—but not modified ones—of the IGT was employed.

At the same time, the following exclusion criteria were adopted: (1) studies adopting tDCS to assess decision making without the IGT; (2) studies applying the IGT without evaluating the influence of the tDCS; (3) book chapters.

### 2.3 Study selection

The screening of relevant articles was conducted firstly by title, keywords, and language and secondly by reading the abstracts and full texts. Specifically, considering the five-stage of the framework of Arksey and O’Malley (2005), the identification stage was performed through the search of specific keywords on bibliographic databases, followed by the screening stage, where the authors selected the articles by a title and language search. Lately, the articles have been analyzed through an abstract screening, followed by a full-text analysis aimed at identifying the suitable papers for the review. In this last stage, to ensure the comparison among the results, the reasons for exclusion were related to a methodological approach, since the papers considered modified versions of the IGT but not the original one were excluded.

Zotero was adopted as reference managing software.

## 3 Results

A total of seven studies have been selected. The articles selected comprehend a time range from 2015 to 2023 (since the literature did not present papers analyzing the effect of tDCS on DM through the adoption of the IGT before the year 2015). All of them adopted a randomized controlled trial methodology. The countries where the studies were conducted are Canada, Italy, Spain, Turkey, and USA. Regarding participants’ information, the lowest average age was 20.75 years (León et al., 2020), while the highest was 63.5 years (Benussi et al., 2017). Four studies assessed a mixed sample of males and females (Ouellet et al., 2015; Benussi et al., 2017; León et al., 2020; Lisoni et al., 2020) while the other three focused on males only (He et al., 2016; Soyata et al., 2019; Salatino et al., 2022). Three studies investigated healthy participants (Ouellet et al., 2015; He et al., 2016; León et al., 2020), while the other four explored the effect of tDCS on the IGT over patients: Specifically, they considered subjects affected by PD (Benussi et al., 2017), Gambling Disorder (GD; Soyata et al., 2019; Salatino et al., 2022), and Borderline Disorder (BPD; Lisoni et al., 2020; for more details, see Table 1).

**Table 1:** Summary of the characteristics of the samples.

Study	Country	Type of the sample	Size of the sample	Sex (male%)	Age (yrs.): mean (sd)	Age range (yrs)	Education (yrs.): mean (sd)
Ouellet et al. (2015)	Canada	Healthy adults	45	35,5%	25.09 (±7.10)	18–60	16.89 (± 2.41)
He et al., 2016	US	Healthy adults	Ex 1: 41 Ex 2: 49 Ex 3: 20	100%	Ex 1: 20.7 (±1.59) Ex 2: 20.5 (±1.63) Ex 3: 19.7 (± 0.92)	Ex 1: 18E–25 Ex 2: 18E–25 Ex 3: 18–21	College students
Benussi et al., 2017	Italy	PD patients	60 20 received A_tDCS, 20 received A_tDCS 20 received sham condition	71.7%	A_tDCS: 63.2 (±9.2) C_tDCS: 62.9 (±10.5) Sham tDCS: 63.5 (±8.6)	Not specified	A_tDCS: 10.3 (±3.4) C_tDCS: 9.5 (±4.6) Sham tDCS: 9.4 (±3.9)
Soyata et al., 2019	Turkey	GD patients	20	100%	37.2 (± 10.3)	18–55	13.4 (± 3.2)
León et al., 2020	Spain	Healthy adults	First phase: 92 Second phase: 61 received A_tDCS or sham condition	First phase: 45.1% Second phase: 44.3%	First phase: 20.76 (± 3.08) Second phase: 20.75 (± 2.82)	Not specified	Not specified
Lisoni et al., 2020	Italy	BPD patients	30 15 received A_tDCS 15 received sham condition	40%	40.3 (± 12.8)	Not specified	12.2 (± 3.3)
Salatino et al., 2022	Italy	GD patients	1	100%	45	Not applicable	21

A_tDCS, anodal transcranial direct current stimulation; BPD, Borderline Personality Disorder; C_tDCS, cathodal transcranial direct current stimulation; Ex, experiment; GD, Gambling disorder; PD, Parkinson’s disease; tDCS, transcranial direct current stimulation; yrs, years.

The improvement in the decisional performance after tDCS stimulation was mainly measured by a higher IGT net score resulted from selecting advantageous decks over the disadvantageous ones. In the selected studies, two main cerebral areas were selected to apply tDCS: the DLPFC (He et al., 2016; Benussi et al., 2017; Soyata et al., 2019; Lisoni et al., 2020; Salatino et al., 2022) and the OFC (Ouellet et al., 2015; León et al., 2020; for more details, see Table 2).

**Table 2:** Summary of the characteristics of tDCS studies.

Study	Study design	Electrodes positions	Polarity	Intensity	Duration	Number of stimulations	Sham condition	DM assessment	Secondary assessment	Aim of the study	Main results
Ouellet et al., 2015	RCT	Received a single 30-min session of either: active anodal left OFC/cathodal right OFC (i.e., “left OFC” group; n ¼ 15) or active anodal right OFC/cathodal left OFC (i.e., “right OFC” group; n ¼ 15) or sham, anodal/cathodal tDCS randomly applied to either the left (n ¼ 7) or to the right (n ¼ 8) OFC (i.e., sham group).	A_tDCS—coupled with contralateral C_tDCS	1.5 mA	30 min	1	Yes	IGT BART	Impulse control: SCWT SST Mood: visual analogue scales Attentional levels: CPT	To examine the influence of tDCS over the OFC on DM modulation and on cognitive impulse control.	Subjects exposed to active A_tDCS (regardless the laterality) showed an increased DM performance and cognitive impulse control. However, there were shown no effects on mood, attentional levels, and motor impulse control.
He et al., 2016	RCT	Ex 1–3: For participants receiving real HD-tDCS with multifocal montage over the left DLPFC, the anodal electrode was placed over F3 (based on the International 10E–20 EEG System) and the four return (cathodal) electrodes were placed over F5, AF3, FC3, and F1, around the active electrode. F3 was used as the anodal electrode location because most previous studies have used this location to stimulate the left DLPFC. Sham stimulation was conducted with the same montage Ex 2: the location of the anodal/sham stimulation was the right DLPFC region. They used the HD-tDCS. Twenty participants (mean age = 19.7; SD = 0.92; years ranging from 18 to 21) who participated in Ex 1 were recruited for this experiment. If they had their HD-tDCS over the left DLPFC in Ex 1, they were assigned to the sham condition for Ex 3 (*n*: 11); if they were in the sham group in Ex 1, they were assigned to the HD-tDCS condition in Ex 3.	A_tDCS coupled with C_tDCS	1.5 mA	20 min	Ex 1: 1 Ex 2: 1 Ex 3: 1	Yes	IGT ITC	Personality trait of impulsivity: BIS	To explore the causal influence of the DLPFC when carrying out the IGT and the ITC task.	Results underlined that tDCS over the left (but not the right) DLPFC improved IGT score and reduced delay discounting rate in the ITC task.
Benussi et al., 2017	RCT	Right DLPFC. The reference electrode was fixed on the contralateral supraorbital area.	Ex 1: C-tDCS Ex 2: A_tDCS	2 mA	10 min	1	Yes	IGT	Not applied.	To investigate the impact of tDCS over the DLPFC in improve gambling behaviour in PD.	C_tDCS over the right DLPFC decreased impulsive and risky DM performance in PD patients on dopam inergic medication. No differences were found between A_tDCS and sham condition results
Soyata et al., 2019	RCT	Either (i) active anodal right/cathodal left (*n* = 10) or (ii) sham anodal right/cathodal left (*n* = 10) tDCS over the DLPFC	A_tDCS—coupled with left C_tDCS	2 mA	20 min	3 per day, every 2 days	Yes	IGT	Abstraction, shifting and categorization: WCST	To examine the influence of tDCS over DLPFC in modulating DM and cognitive flexibility in GD patients.	Results highlighted that tDCS has enhanced DM performance and cognitive flexibility in GD.
León et al., 2020	RCT	Right OFC	A_tDCS (controlateral with trapezium)	1.5 mA	20 min	1	Yes	IGT	Response inhibition: SST	To analyze for the first time the interaction between sex and tDCS in DM performance.	In the first part of the study, data indicated that men registered a better performance than women in the IGT. However, in the second part the stimulation influenced the IGT performance according to the sex: anodal tDCS increased the IGT performance in women, while in men the stimulation did not have an impact.
Lisoni et al., 2020	RCT	Electrodes were placed with a bipolar-balanced montage with anode on the right DLPFC (F4) and cathode on the left DLPFC (F3) according to EEG 10-20 system.	Right A_tDCS coupled with left C_tDCS	2 mA	20 min	15	Yes	IGT	Impulsivity: BIS-11; Aggressiveness: BP-AQ Emotion regulation: DERS Depression: HDRS BDI Anxiety levels: HAM-A IDA Craving: VAS	To investigate the influence of tDCS over the core dimensions (impulsivity, aggression, affective dysregulation) of BPD, decision making process and substances craving.	The application of bilateral tDCS seems to improve core dimensions of BPD (mainly impulsivity and aggression) probably by restoring prefrontal activity. Craving intensity was reduced only in the active-tDCS sample. Both groups (active and sham) showed improvements in the affective dysregulation dimension and anxious and depressive symptoms. Impulsivity and aggression measures were significantly reduced only in patients treated with active-tDCS. Decision-making process was influenced by the active current. Craving intensity was reduced only in the active-tDCS sample.
Salatino et al., 2022	RCT	Bilateral DLPFC	Right A_tDCS—coupled with left C_tDCS	1 mA	20 min	6	No	IGT BART	Anxiety levels, impulsivity in everyday life and quality of life: HAM-D, BIS-11 SF-36 Global cognitive functioning: MoCA Specific gambling tests: SOGS CPGI Impulse control: Go/No go task.	To determine the effectiveness of a novel low dose tDCS on DLPFC in a GD patient.	The results underlined an improvement of impulsivity control, DM, and cognitive functioning in BART but not in IGT after tDCS sessions. Those findings propose that: low doses of right anodal/left cathodal tDCS to DLPFC may improve gambling behaviour future research should investigate the effects of this tDCS polarity on the patient’s emotional state.

A_tDCS, anodal transcranial direct current stimulation; BIS-11, Barratt Impulsiveness scale; BART, Balloon Analogue Risk Task; BIS, Barratt Impulsivity Scale; BDI, Beck Depression Inventory; BP-AQ, Aggression Questionaire; CPGI, Canadian Problem Gambling Index; CPT, Continuous Performance Task; C_tDCS, cathodal transcranial direct current stimulation; DERS, Difficulties in Emotion Regulation Scale; DLPFC, dorsolateral prefrontal cortex; DM, decision making; Ex, experiment; GD, Gambling Disorder; HAM-A, Hamilton Anxiety Rating Scale; HAM-D, Hamilton Rating Scale for Depression; HDRS, Hamilton Depression Rating Scale; HD-tDCS, High-definition transcranial Direct Current Stimulation; IDA, Irritability-Depression-Anxiety Scale; IGT, Iowa Gambling Task; ITC, Inter-Temporal Choice task; MoCA, Montreal Cognitive Assessment; mPFC, medial prefrontal cortex; OFC, orbifrontal cortex; PD, Parkinson’s Disease; RCT, randomized controlled trial; SCWT, Stroop Colour-Word Test; SF -36, Short Form Test–36; SOGS, South Oaks Gambling Screen; SST, Stop-Signal Task; VAS, Visual analogic scales; WCST, Wisconsin Card Sorting Test.

Overall, in the seven studies selected no significant difference regarding the effects of the tDCS was identified depending on the duration of the stimulation. Moreover, the most commonly adopted modality in the selected studies was anodal tDCS coupled with contralateral cathodal tDCS, although two of them used cathodal tDCS (Benussi et al., 2017; Soyata et al., 2019). Only one study assessed subjects just with the anodal modality (León et al., 2020), where the reference electrode was located over the contralateral trapezium and only one study (Benussi et al., 2017) investigated and reported disparities regarding the higher effectiveness of the application of cathodal tDCS rather than anodal.

### 3.1 Healthy subjects

With regards to healthy subjects, He et al. (2016) were the only authors stimulating DLPFC. The researchers conducted three experiments. In experiment 1 a group of subjects was exposed to tDCS (active or sham condition) over the left DLPFC, while in experiment 2 participants were assessed with tDCS (active or sham condition) on the right DLPFC. An additional experiment was designed, where a subset of the first group was recruited and assigned to the condition they were not in the first experiment. Therefore, if the subjects were assigned to the sham condition in experiment 1, in experiment 3 they had been affiliated with the tDCS condition over the left DLPFC. Likewise, if the participants were assigned to the stimulation condition in experiment 1, they were allocated in the sham condition in experiment 3. Overall, the study recorded an improvement in the IGT scores over the left (but not the right) DLPFC after the tDCS session. Moreover, the authors also controlled possible order effects without finding any significant main or interaction effect.

The other two studies on healthy subjects stimulating the OFC (Ouellet et al., 2015; León et al., 2020) reported an increased IGT performance under ambiguous and risky conditions and a higher cognitive impulse control. In detail, Ouellet and colleagues (Ouellet et al., 2015) documented that tDCS on either left or right OFC enhanced DM and cognitive impulse control in the IGT. Other cognitive abilities assessed (i.e., attentional levels, mood, and motor impulse control) did not improve. León and colleagues (León et al., 2020) focused on the interaction between sex (as the specific biological attribute correlated with physical and physiological features) and tDCS over the right OFC in the IGT performance. The findings outlined that, in the first phase of the study where DM was assessed only through the IGT, men registered an overall better performance than women. In the second phase, all subjects were stimulated through tDCS and then they proceeded in performing again the IGT. Results indicated that the stimulation influenced the IGT performance according to the sex since anodal tDCS increased the IGT performance in women but did not impact men’s outcomes. Regarding other variables, the authors did not identify any effect of tDCS nor sex in response inhibition (Logan et al., 1984).

### 3.2 Patients

Concerning pathological populations tested through tDCS, all four studies focused on the DLPFC. An improvement in the IGT performance was observed in either GD, PD, and BPD patients. When analyzing GD patients, in Soyata et al. (2019), the application of a bilateral stimulation on DLPFC resulted in an improvement in IGT performance, cognitive flexibility, and impulsivity control. Meanwhile, in Salatino et al. (2022), the patient did not significantly improve in the IGT.

When considering patients affected by PD on dopaminergic medication, Benussi et al. (2017) found an increased IGT performance in the cathodal condition when compared to sham one.

Finally, analyzing BPD patients, Lisoni et al. (2020) reported that the DM process was influenced by bilateral tDCS over the DLPFC, as the active tDCS sample showed improved performance in the IGT as compared to sham patients. Specifically, the first group demonstrated a less risky and more cautious behavior.

## 4 Discussion

The present findings suggest four main points of discussion, that involve (i) the potential different effects of the polarity application (anodal or cathodal) and the position; (ii) the importance of the DLPFC and OFC in the DM process; (iii) the possible influence of individual variables on the impact of tDCS on the IGT performances, such as sex; (iv) the importance to delve into the learning process within the enhancement of DM abilities during the IGT performance. The authors have deepened each point and provided potential insights for future analysis.

### 4.1 Electrode polarity and position

The difference between anodal-excitation and cathodal-inhibition have been investigated only in one study, considering PD patients (who regularly took dopaminergic medications and were tested in the “on” phase) by Benussi et al. (2017). The authors found an improvement in the IGT performance only by cathodal tDCS over the right DLPFC. This can support the existence of a delicate balance between the affective dimension related to possible rewards and the cognitive one, that in PD patients may be altered by dopamine medications (e.g., Colautti et al., 2024). In fact, it is assumed that exogenous dopamine may contribute to develop a tendency toward risky choices, where patients’ decisions are based more on rewarding consequences rather than potential losses. Such a behaviour has been linked to a decreased sensitivity to losses or to possible impairments in anticipating the unrewarding consequences (Kobayakawa et al., 2010; Colautti et al., 2021, 2023), according to the dopamine overdose hypothesis (Gotham et al., 1986; Cools et al., 2022). In this way, cathodal tDCS over the right DLPFC can increase cognitive control, and thus reduces impulsivity and risky (but more rewarding) choices in PD, possibly by decreasing the overdrive in the frontostriatal circuits biased by dopaminergic medications, connected to prefrontal areas.

Focusing on healthy subjects, no study investigated the difference of tDCS polarity: both Ouellet et al. (2015) and He et al. (2016) applied anodal tDCS coupled with contralateral cathodal tDCS over the OFC and DLPFC, respectively, and they both reported DM improvements. In this way, it may be interesting if further studies will explore also in healthy subjects possible differences in DM improvement by applying different polarity.

Regarding the considered cortical regions, He et al. (2016) reported that tDCS improved the IGT scores in healthy subjects when applied over the left (but not the right) DLPFC. On the other hand, considering PD patients by Benussi et al. (2017) and GD patients by Soyata et al. (2019) results showed positive effects on DM when electrodes were applied on the right DLPFC and either on the left or right DLPFC respectively, confirming the importance of considering the specific characteristics of the different samples and the possible underlying pathophysiological process. Concerning healthy samples, no differences in the electrodes’ position were highlighted considering tDCS application on the OFC. Specifically, León et al. (2020) reported positive improvements in DM after stimulating the right OFC, while Ouellet et al. (2015) indicated enhanced DM abilities after stimulating either the left or right OFC. Therefore, further research may better explain the influence of the left or right electrode positioning on both the DLPFC and the OFC whenever examining the brain regions involved in risky and ambiguous DM.

### 4.2 The importance of the DLPFC and OFC in the DM process

The importance of the DLPFC and the OFC in risky and ambiguous DM seems to be sustained (Ward, 2019) considering that the majority of the studies considered supported the pivotal role of the DLPFC in inhibitory control (Oldrati et al., 2016; Nejati et al., 2018; Colombo et al., 2020; Salehinejad et al., 2021) and the relevance of OFC, probably contributing to emotion regulation (Ochsner and Gross, 2005; Nejati et al., 2018). In fact, as argued by Yang et al. (2017), who stimulated both the DLPFC and the OFC with tDCS adopting a “risk/ambiguity decision-making task,” DM processes under risk and ambiguity may involve distinct circuits and processes. The authors suggested that the DLPFC may be primarily involved in decisions under risk whereas the OFC is associated to ambiguity. This supports the possible involvement of the DLPFC in cognitive control and in abilities encompassing cold EFs (since risky DM requires a careful balance between potential results and the probabilities of occurrence of each possible outcome) and the OFC in more emotional contexts and abilities encompassing hot EFs (as ambiguous DM includes a factor of uncertainty; Bechara et al., 2000; Fellows and Farah, 2007; Pessoa, 2009; Chase and Clark, 2010; Nejati et al., 2018). In this way, the involvement of cold and hot cognition (and in particular of EFs; For more details: see the Introduction section) can be supported by these results, confirming the interaction between the two systems throughout the DM process. In fact, consistently to Colombo et al. (2020), when the DLPFC is inhibited and the OFC is enhanced, people favour faster and impulsive choices aimed at obtaining an immediate reward; Conversely, when DLPFC’s activity is enhanced, individuals undertake a decisional process that is sustained by cold cognition and consequently they are less impulsive and choose long-term delayed rewards (Nejati et al., 2018).

#### 4.2.1 A brief focus on DM in pathological conditions and the effects of tDCS on specific brain areas in pathological conditions

The stimulation of DLPFC and OFC in patients produced outcomes depending on the characteristics of the disorder.

##### 4.2.1.1 GD

Concerning GD patients, it was found that tDCS sessions focused on right anodal tDCS coupled with left cathodal tDCS on the DLPFC can enhance DM performance and cognitive flexibility in the study conducted by Soyata et al. (2019), while no improvements in DM assessed through IGT were reported by Salatino et al. (2022) after tDCS, even if an enhancement in the decisional abilities and risk-taking was recorded adopting another DM task, the Balloon Analogue Risk Task (BART). Unfortunately, Salatino and colleagues’ study involved only one patient and it is possible that individual characteristics may have biased the result However, further studies are needed, to better highlight the contribution of the DLPFC in the IGT (O'Keeffe, 2009; Li et al., 2010; Obeso et al., 2021) sustaining the top-down regulation in DM processes (Fleck et al., 2006; Alizadehgoradel et al., 2020; Zhao et al., 2021).

Overall, with regards to tDCS, Salatino et al. (2022) confirmed the improvements in general cognitive functioning and DM in the GD patient. This result might be explained by the neurophysiological alteration and abnormal dopaminergic activity mentioned before: Neuroimaging studies reported that GD patients show reduced responses in the ventral striatum and VMPFC, linked to hot EFs, during reward processing and that this effect is correlated with gambling severity (Reuter et al., 2005). Moreover, they demonstrated hypoactivity in the VMPFC in affective switching paradigm (de Ruiter et al., 2009). Finally, it was reported that closed losses leading to dopaminergic midbrain activity were positively correlated with GD severity due to the abnormal reward system activity (Chase and Clark, 2010). Accordingly, Linnet et al. (2011) showed that dopamine release in the ventral striatum was associated with adaptive behaviour in healthy individuals and maladaptive behaviour in GD sufferers during the IGT, who were elaborating the dopaminergic “reward” from ambiguity, leading to a reinforcement of risk maladaptive behaviour. These results demonstrated that GD patients had significantly higher excitement and suggested that they may suffer from a dopaminergic “double deficit” condition (Koob and Volkow, 2016), where dopamine release is associated with both impaired gambling behaviour and increased excitement levels and that both factors may contribute to the GD (Linnet, 2013).

Analyses of task reaction times showed faster responding and lower response-shifting after losses in GD patients (Goudriaan et al., 2005), supporting the hypothesis of a diminished reward and punishment sensitivity or reduced feedback processing following a penalty. Indeed, the increasingly selections of disadvantageous decks demonstrate less sensitivity to losses, but also an increased reward sensitivity due to the higher immediate win amounts (Balodis, 2020). Therefore, the application of anodal tDCS over the right DLPFC might have increased cognitive control and the ability to use feedback, updating, and set-shifting, which are essential abilities within the cold EFs (Friedman and Robbins, 2022), among which the DLPFC has a pivotal role (Krain et al., 2006). Moreover, the excitatory effect of anodal tDCS improved the pathological hypo-reward sensitivity involving the dopaminergic circuits.

##### 4.2.1.2 PD

Concerning PD, tDCS sessions focused on cathodal right DLPFC promoted a decreased impulsivity, and so fewer risky choices (Benussi et al., 2017), supporting the notion of a better elaboration of wins and losses. As previously discussed, findings from this study focused on PD patients who regularly took dopaminergic medications suggested the existence of a delicate balance between the affective dimension related to possible rewards and the cognitive one, figuring out a tendency toward risky choices in such patients (e.g., Colautti et al., 2024).

Further research should explore whether the same enhanced performances in the IGT after tDCS are achieved by PD patients who are not on dopaminergic medication, as evidence shows that PD patients during “off” conditions present a different sensitivity toward reward and losses by decreasing risky choices (e.g., Cools et al., 2022).

##### 4.2.1.3 BPD

Concerning the BPD condition, tDCS sessions focused on bilateral right anodal and inhibitory left cathodal over the DLPFC produced an improvement in DM (Lisoni et al., 2020). However, according to Lisoni et al. (2020), this positive effect was only partially attributable to tDCS, since the analysis of the interaction between time and treatment revealed just a statistical trend (*p* = 0.07), which could be explained by “the small sample number of participants rather than a failed engagement/modulation of OFC during the task” (p. 8). Nevertheless, they also supported the consistency of these positive results with other studies targeting the right DLPFC with anodal tDCS in other pathological conditions where DM impairments can occur (e.g., substance abusers: Gorini et al., 2014; impulsive veterans: Gilmore et al., 2018). Overall, the results highlight the neurobiological substrates of impulsivity regulation involved in the prefrontal cortex with its component domains (including cognitive control, planning, risk-taking, and delay discounting) and demonstrate that tDCS over the right DLPFC could improve behavioural and cognitive impulsive manifestations and aggression in BPD, probably by restoring the prefrontal activity on subcortical structures (Lisoni et al., 2020).

These findings are supported by the literature, which reports the significant influence of the DLPFC in regulating behavioural control (New et al., 2009; Coma Gonzalez et al., 2024) and its key role, working together with the OFC, in impulsivity regulation (Chanen et al., 2008; Sabbah et al., 2024). Specifically, it was reported that enhanced impulsiveness and aggression are related to the alteration of the DLPFC functioning in BPD patients and may therefore facilitate the development of impulsive behaviours and aggressiveness (Sala et al., 2011). Likewise, in a PET study, New et al. (2009) showed that the DLPFC in BPD subjects did not activate when assessing aggression traits. Therefore, since there are no medications yet approved to treat BPD and the therapeutic procedure is mainly based on a symptom-based approach (Vita et al., 2011; Baumann and Herpertz, 2022), which are promising but not definitive because they do not modify the neurobiological substrates of the disorder, the authors suggest tDCS as a harmless, highly-compatible, non-invasive neurostimulation technique able to induce neuroplasticity (Jamil et al., 2017) and to modulate cognition in BPD (Lisoni et al., 2020).

### 4.3 Sex as a variable to consider

Sex was not fully considered when evaluating the elements impacting the effectiveness of tDCS on the IGT performance. In particular, only one study assessed the effects of sex (León et al., 2020), while all the other studies did not consider it as an influent element to investigate. As reported in the literature, males and females exhibit differences in the IGT (Flores-Torres et al., 2023), where males usually select options that yield larger long-term rewards compared to females (Cornwall et al., 2018; Overman, 2004; van den Bos et al., 2013), are less susceptible to unexpected losses in a string of wins, leading to change the strategy (Brand et al., 2007), and learn faster which are the most beneficial decks to take more advantageous choices on the IGT (Cornwall et al., 2018; Garrido‐Chaves et al., 2021). So, possible interesting results may be undermined by considering both men and women together, without further investigating possible sex differences. Therefore, further studies should consider the influence of sex in the IGT especially when applying tDCS.

### 4.4 The learning process

Only two studies investigated the DM performance over the learning process (He et al., 2016; León et al., 2020) throughout the different IGT trials, dividing them into blocks, confirming that all participants performed better in post-intervention session regardless of sex and stimulation by means of learning effects (León et al., 2020) and that, compared to a sham group, the faster learning occurred for the IGT scores in the trials 41–60 (He et al., 2016). In all other studies, the analysis just involved a comparison between the net score recorded before the tDCS and the potential improvement achieved in a second moment after the tDCS session (by the total net score).

Another article has deepened the investigation of the learning process during the IGT (Wang et al., 2017) by assessing a sample composed of 34 young male students performing the decisional task using high-definition tDCS (HD-tDCS). The participants were divided into three groups. The first one received cathodal inhibitory tDCS over the rostral anterior cingulate cortex (rACC); The second one received inhibitory tDCS over the posterior cingulate cortex (PCC); The third one received sham tDCS over the primary motor cortex. The adoption of a different type of tDCS and a modified version of IGT are the reasons of the exclusion of this paper from the screening selection of the current review. However, it is important to highlight the findings since this paper is one of the few investigating the learning process through tDCS and IGT and the brain areas involved. Compared to previous studies, which adopted functional magnetic resonance imaging (fMRI) data, the authors decided to implement HD-tDCS to alter the activation in updating the prediction-related brain regions, since fMRI can provide correlation but is not able to readily demonstrate necessity (Wang et al., 2014). The authors aimed at investigating the prediction error (PE), which is stated to arise when there is a difference between expected and actual outcomes, and the learning process during risky DM assessed throughout the IGT (Lak et al., 2014).

Learning from PE for updating the prediction in reinforcement learning has been long investigated in the literature and many results supported the presence of PE-related brain activations (e.g., the dopamine system including the striatum, the prefrontal cortex, etc.). However most previous studies have only focused on general neural coding of PE and the related behavior, while not comparing the different kinds of PE processing directly (Gläscher et al., 2010; Hollerman and Schultz, 1998; Robinson et al., 2013). Therefore, the neural mechanism that specifically underlies learning from PE for updating the prediction during the DM still needs to be investigated (Bach and Dolan, 2012). The net score was calculated for each of the nine blocks (each block involving 20 trials) and differences between the sham condition and the tDCS condition were analyzed. It was found that electric brain stimulation lowered the performance in the decisional task. The study confirmed that PEs were used for updating the prediction in the IGT throughout a learning process occurring in the different trials. Specifically, it was highlighted that the rACC, the ventral medial prefrontal cortex (vmPFC), and the PCC were activated during the task and were related to both reward and risk PE and that were modulated by uncertainty. Overall, the findings supported the presence of a neural circuit of PE processing and suggested that the rACC/vmPFC and the PCC play a key role in updating the prediction through PE processing during DM (Wang et al., 2017).

Considering the preliminary findings reported by the literature regarding the learning process in the IGT, which is pivotal in such a task and in everyday risky situations, it would be useful that further studies should investigate such a process dividing the net IGT scores into blocks (in addition to considering the total net score), to better study and deepen our understanding on how tDCS can help support this learning process. Moreover, it would be possible to better investigate the possible shift from DM under ambiguity to DM under risk as feedback is provided along the task, exploring in a deeper way how tDCS improves the DM performance along the IGT.

### 4.5 Limitations

Some limitations were identified in the present scoping review, mostly due to the characteristics of the studies analyzed. Only a few studies investigated the effects of tDCS on the IGT in different samples (healthy adults, GD subjects, PD patients). This limitation undermines possible comparisons between samples regarding the different effects that tDCS can have on the decisional process, exploring whether specific target populations might benefit more from tDCS rather than others.

Another element to consider is the heterogeneity of the research designs adopted by the studies, involving the number of tDCS sessions scheduled in each study, the electrodes positions, their polarity (anodal or cathodal), the number, the sex differences (male or female), and the type (healthy subjects or patients) of participants. Therefore, further studies are needed to confirm the findings so far and to deepen the uncovered issues.

Furthermore, this paper was focused only on tDCS effects on DM, even if other techniques can be used, for instance transcranial magnetic stimulation (TMS) or transcranial Alternating Current Stimulation (tACS). We decided to specifically focus on tDCS because it is becoming an increasingly popular technique in clinical setting, it is less expensive than TMS, and does not typically cause serious adverse effects (Elder and Taylor, 2014; Stagg and Nitsche, 2011). On the other side, tACS shares the same settings of tDCS in terms of device and montage but differs in terms of current flow wave-form delivered through the scalp, since it delivers electrical oscillatory currents at different frequency ranges according to the operator’s demands (Feurra et al., 2012). Recent studies have shown that tACS may boost brain activity related to different functions (Yaple et al., 2017), however others investigating the effect of tACS on DM under ambiguity showed no direct effect of tACS on exploration behaviour and general risk-taking (Wischnewski and Compen, 2022). Moreover, as shown by the review of the screened articles, there are still very few studies investigating the use of tACS for cognitive processes underlying DM (Brunyé, 2021).

The authors of the present review decided to focus on a single methodology as tDCS to ensure comparisons among the results of the studies considered. Anyway, this exploratory contribution may provide support for further studies investigating DM processes also considering other techniques.

### 4.6 Conclusion

The present scoping review examined the studies conducted to deepen the effects of tDCS over DM involving ambiguous and risky conditions, specifically assessed through the IGT. Overall, the findings support the notion that tDCS can enhance the overall performance in the IGT in both healthy adults and patients affected by GD, PD, and BPD.

The results pointed out that it is possible to improve the IGT performance after the stimulation of the DLPFC, confirming the crucial role of this area in decisional processes (Luo et al., 2017; Ota et al., 2019; Colautti et al., 2022). The DLPFC is assumed to mainly be involved in “cold” cognition and metacognitive control—which are goal-directed processes pivotal in everyday life—and to be implied, together with the OFC, in emotion regulation, especially in those situations characterized by high emotional arousal (Chan et al., 2008; Nejati et al., 2018). Therefore, at least in healthy subjects, anodal tDCS, providing a neuron-excitatory effect over the DLPFC, would enhance cognition-based skills such as problem-solving abilities, planning, and working memory (Alizadehgoradel et al., 2020), and processing ambiguous and risky decisions. Conversely, the OFC, the other neural region targeted in the analysed studies, is highly connected to dopaminergic and limbic circuits and is assumed to underlie to a greater extent the so-called “hot” cognition, involved in contexts characterized by emotions, incentives, reward processing, and possible conflict between an immediate reward and a long-term benefits (Zelazo et al., 2014; Nejati et al., 2018). This region is also highly interconnected with the DLPFC and patients with OFC lesions are found to have a higher level of impulsivity, being unable to shift towards less risky choices in DM despite negative outcomes and making riskier choices compared both to healthy controls and those with damages in other brain regions (Ouellet et al., 2015). Therefore, the OFC might be considered as a crucial connecting region between “cold” and “hot” cognitive control, as showed by studies about emotion and reward processing, feedback learning, and the inhibition of automatic and instinctive responses (Rushworth et al., 2007; Liu et al., 2009; Pessoa, 2009). Such an assumption can be supported by the studies analysed in the present review (Ouellet et al., 2015; León et al., 2020) that highlighted enhanced abilities to process long-term reward over the immediate satisfaction in the IGT after receiving tDCS.

It is important to continue to investigate the role and implication of the DLPFC and OFC in DM and to deepen their involvement. This can be crucial to (i) shed light on how clinical conditions affecting brain regions that are crucial for DM can negatively impact patients’ choices, identifying individuals at high risk of developing severe symptoms or complications from neurological or psychiatric conditions and preventing possible negative consequences derive from impaired decisional abilities; (ii) design protocols adopting tDCS as a treatment approach to maintain a significant level of therapeutic adherence, individual autonomy, and wellbeing.

## Data Availability

The original contributions presented in the study are included in the article/supplementary material, further inquiries can be directed to the corresponding author.
